# Stable Isotopes Provide Insight into Population Structure and Segregation in Eastern North Atlantic Sperm Whales

**DOI:** 10.1371/journal.pone.0082398

**Published:** 2013-12-06

**Authors:** Asunción Borrell, Adriana Velásquez Vacca, Ana M. Pinela, Carl Kinze, Christina H. Lockyer, Morgana Vighi, Alex Aguilar

**Affiliations:** 1 Department of Animal Biology, Faculty of Biology and Biodiversity Research Institute (IRBio), University of Barcelona, Barcelona, Spain; 2 Zoological Museum, University of Copenhagen, Copenhagen, Denmark; 3 Age Dynamics, Konhgens Lyngby, Denmark; Hawaii Pacific University, United States of America

## Abstract

In pelagic species inhabiting large oceans, genetic differentiation tends to be mild and populations devoid of structure. However, large cetaceans have provided many examples of structuring. Here we investigate whether the sperm whale, a pelagic species with large population sizes and reputedly highly mobile, shows indication of structuring in the eastern North Atlantic, an ocean basin in which a single population is believed to occur. To do so, we examined stable isotope values in sequential growth layer groups of teeth from individuals sampled in Denmark and NW Spain. In each layer we measured oxygen- isotope ratios (δ^18^O) in the inorganic component (hydroxyapatite), and nitrogen and carbon isotope ratios (δ^15^N: δ^13^C) in the organic component (primarily collagenous). We found significant differences between Denmark and NW Spain in δ^15^N and δ^18^O values in the layer deposited at age 3, considered to be the one best representing the baseline of the breeding ground, in δ^15^N, δ^13^C and δ^18^O values in the period up to age 20, and in the ontogenetic variation of δ^15^N and δ^18^O values. These differences evidence that diet composition, use of habitat and/or migratory destinations are dissimilar between whales from the two regions and suggest that the North Atlantic population of sperm whales is more structured than traditionally accepted.

## Introduction

In the main oceans of the world, the absence of geographical barriers permits pelagic species to occupy vast geographic ranges inside which they often engage in long migratory movements. This result in high population sizes which, together with the high mobility of many of the species, favours mild genetic differentiation and consequent absence of structuring [1,2]. However, among large cetaceans there have been recurrent examples of structuring and segregation within otherwise large and apparently homogeneous areas of distribution, probably as a consequence of environmental patchiness, the evolutionary history of populations, behavioural traits such as resource partitioning and social affiliations, and cultural or maternally transmitted fidelity of migratory destinations [3-6]. 

Sperm whales are migratory odontocetes situated at the top of the food web that inhabit mesopelagic marine ecosystems. They are slow-growing animals that live as long as 70 years [7] and their diet varies with sex, age and geographical region [8-10]. Individuals from different segments of the population establish long-lasting bonds and group into social units which often show allopatric geographic distributions. Thus, adult females with their offspring and juveniles form the matrilineally based social units, the so-called primary social groups, which occupy tropical and temperate waters of all large oceans. When reaching an age between 4 and 21 yr males leave the breeding units to join the bachelor schools, structures which are thus formed by males in their teens and twenties that are not considered to be socially mature. From their mid-twenties, males may start migrating from low to high latitudes. Males in their forties and older are usually solitary and tend to move to higher latitudes where they remain until returning on an unknown schedule to lower latitudes to meet the primary social groups and reproduce [11-15]. However, the frequency and duration of the large-scale latitudinal migrations a well as the extent of the geographical segregation are still poorly understood.

Sperm whales have a global distribution and sustain significant populations in all large oceans of the world. In the North Atlantic, since long ago the International Whaling Commission (IWC) has recognized the existence of only one single stock of sperm whales based on analyses of commercial whaling data, distribution of sightings, evidences of long-range movements from mark-recaptures within the North Atlantic and similarities between different locations in mortality rates and trends in body lengths [16-18]. For instance, harpoons or harpoon fragments from the Azores were found, years after their deployment, in the bodies of whales killed off Iceland and NW Spain, indicating long-range movements across the eastern North Atlantic [19-21]. Moreover, the case for the existence of a single ocean-wide population received later support by studies showing absence of subpopulation structure in other large oceans [22] and by worldwide genetic studies that were unable to find clearly distinct subpopulations at less than an ocean basin level [23,24]. However, some doubts have persisted not only because data from historical pelagic exploitation in the North Atlantic showed clearly delineated whaling grounds suggestive of significant patchiness in the species distribution [25,26] but also because genetic studies have revealed differences between sperm whales from the Mediterranean Sea and their neighbouring eastern North Atlantic counterparts, thus indicating that subpopulation structuring may indeed occur [27].

Some light into this subject can be shed by the use of stable isotopes, chemical markers that in the last decades have become a technique of choice to track movement and to define geographic occupancy in many species [28-32], including sperm whales [33,34]. Due to differences in the isotope elemental mass, the isotope composition of animal tissues reflect a combination of the parameters of the environment in which they live, such as temperature, salinity and productivity, in addition to biological parameters characteristic to the individuals involved, such as dietary preferences, trophic relationships, physiology and behaviour [35]. Among the various body tissues, tooth dentine, which is made of proteins and hydroxyapatite, is particularly useful because it is deposited in the crown and root of the teeth during the entire life of the animal without existence of turnover [36]. As a consequence, the growth layers that form each year provide material for the reconstruction of ontogenetic time series of isotopic values [37]. The stable isotopes of carbon (δ^13^C) and nitrogen (δ^15^N) are the most commonly used in this type of studies, but the stable isotopes of other elements, such as oxygen (δ^18^O) are also seldom used to provide further resolution [38]. In marine mammals, these three elements are incorporated mostly through dietary protein and ingested water, and their isotopes are subject to different and predictable changes when being transferred through the ecosystem, thus allowing tracing of animal’s movements and foraging behaviour [30,37,39-42].

In this study, stable isotope signatures of carbon, nitrogen and oxygen were studied in the dentinal growth layer groups (annual groups of laminae) of nine sperm whales sampled at two different geographical regions in the North Atlantic (NW Spain and Denmark) to investigate potential differences in the deposited isotopic signatures, particularly in the first years of life, which can be indicative of different reproductive regions (breeding units). 

## Materials and Methods

### Ethics Statement

The sperm whale teeth used for this study were obtained from the biological tissue bank of the University of Barcelona (BMA Tissue Bank) and originated either from commercial fisheries (samples from NW Spain) or from naturally stranded individuals (samples from Denmark). No specific approval is required in Spain to undertake research on samples supplied by official channels and coming from commercial fisheries or stranded individuals.

### Study site and sampling

**Figure 1:**
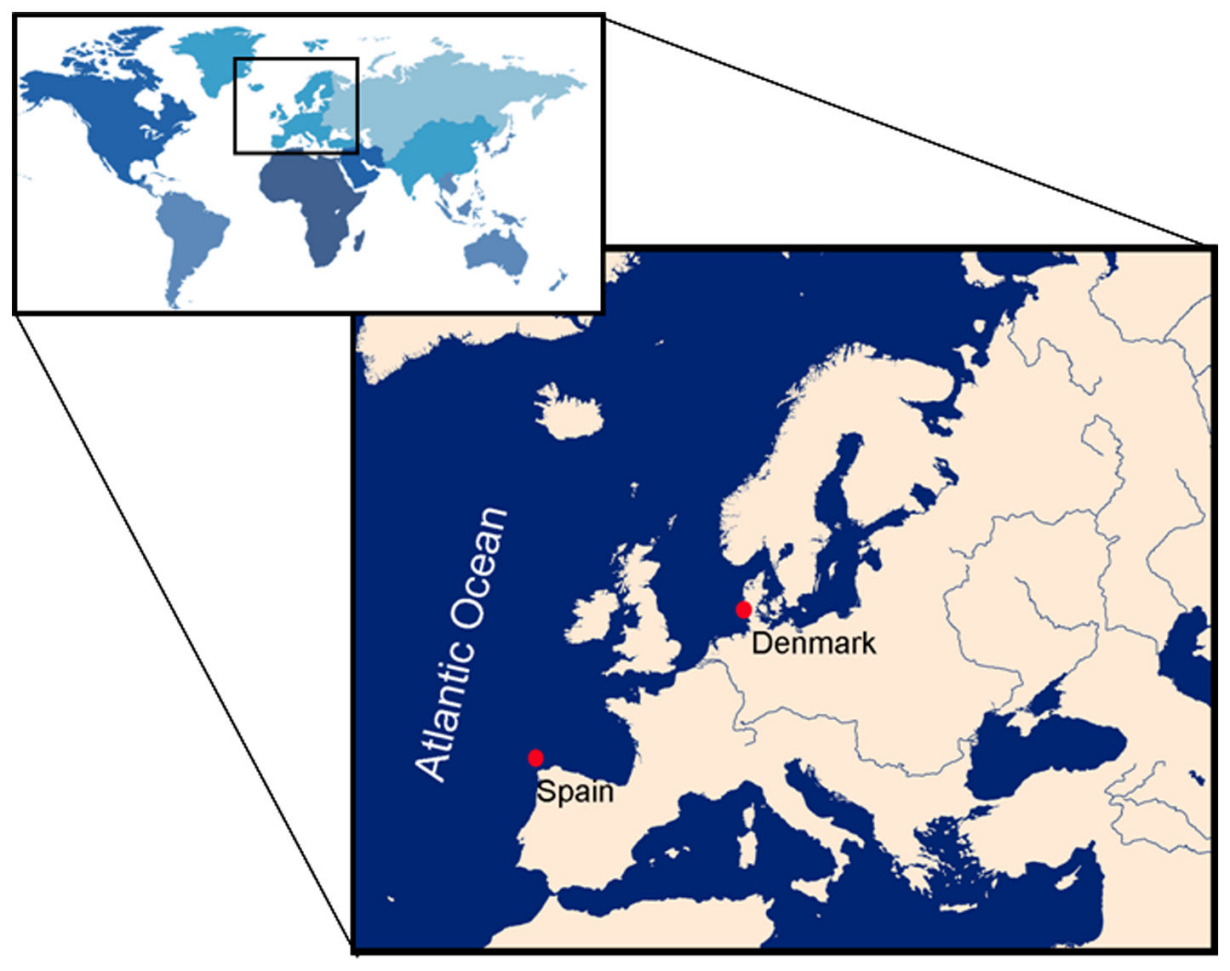
Location of sampling sites.

Five individuals were caught off northwestern Spain (Galicia, thereafter NW Spain) and flensed at the whaling factories of the company Industria Ballenera S. A. during the summer whaling seasons of 1978 and 1980 under catch quotes issued by the International Whaling Commission. Four individuals were sampled from two mass strandings that took place on the northern shores of the island of Rømø in the southwestern Danish North Sea coast [43] (Figure 1). In both regions the whales were measured and sexed (Table 1), teeth were extracted and preserved at the biological tissue bank of the University of Barcelona (BMA Tissue Bank).

**Table 1:** Information on the sperm whales used in the study.

**ID**	**Sex**	**Body length (m)**	**Region**	**Source**	**Year of sampling**	**Estimated age (years)**	**Number of samples analysed**
A	Male	12.2	NW Spain	Captured	1980	16	9
B	Male	11.6	NW Spain	Captured	1980	18	10
C	Female	10.9	NW Spain	Captured	1978	18	9
D	Female	9.5	NW Spain	Captured	1978	20	9
E	Female	10.1	NW Spain	Captured	1978	13	6
F	Male	12.6	Denmark	Stranded	1996	24	7
G	Male	13.2	Denmark	Stranded	1996	27	8
H	Male	12.9	Denmark	Stranded	1996	22	7
I	Male	14.0	Denmark	Stranded	1997	55	12

### Tooth sampling

Teeth were cut in half, with one half being used for age determination and the other one to obtain the dentine samples for isotope analysis. For the latter purpose, circa 20 mg of dentine powder from each growth layer group (GLG) was drilled off using a Dremel™ driller. The powder was divided into two subsamples, one for carbon and nitrogen determination and one for oxygen determination. 

### Age estimation

The age of individuals was estimated by counting dentinal GLGs. Independent age estimates were obtained by two different readers (AA and CL) to account for subjectivity. GLGs were interpreted as those identified in the report of the Workshop on Age Determination of Odontocete Cetaceans and Sirenians [44] as “a repeating or semi-repeating pattern of adjacent groups of incremental growth layers within the dentine which is defined as a countable unit involving a change from a ridge to groove”. The neonatal line was not included in the total number of GLGs. The final age estimate for each individual was determined as the mean of all counts (pooling the counts of the two readers). 

To compare the isotopic signal while individuals were still at the breeding grounds we compared values in GLGs formed during the third year of life. This age was chosen to prevent isotope signature variations caused by: i) lactation, because δ^15^N in mammals enriches during maternal nourishment [45-49] and sperm whale calves suckle for approximately 14-16 months [8]; and ii) migration, because juveniles leave their breeding units at an age of 4-21 years [12,50] so they are expected to start changing their tissue isotopic values thereafter. However, in individual I from Denmark the distal tip of the tooth was worn and the GLG corresponding to the fifth year was the first that could be properly sampled and, because age 5 is still at the bottom of the range of first migration, the isotope values obtained from that GLG were used to characterise the breeding ground signature of that particular individual. 

### Stable isotopes

#### Carbon and nitrogen

When analysing teeth, some authors carry out a preventive demineralisation to eliminate the inorganic carbon by treating the teeth with either a 0.5 M or a 1 M hydrochloric acid (HCl) solution [33,34,37,51]. However, concern has been expressed that such treatment could adversely affect the nitrogen isotopic signature [52]. Taking this into account, we conducted a test on a subset of the samples (n = 20) and found that neither δ^13^C nor δ^15^N values differed between demineralised and untreated samples, so subsequent analyses were carried out without demineralisation.

Approximately 1 mg of the powdered sample was weighed in tin capsules, automatically-loaded, and combusted at 1000 °C to be analysed in a continuous flow isotope ratio mass spectrometer (Flash 1112 IRMS Delta C Series EA Thermo Finnigan). Standards for ^13^C and ^15^N were the Vienna Pee Dee Belemnite limestone (V-PDB) standard and atmospheric nitrogen (air), respectively. International isotope secondary standards of known ^13^C/^12^C ratios in relation to V-PDB, namely: polyethylene (IAEA CH_7_; δ ^13^C = -31.8‰), graphite (USGS24; δ^13^C = -16.1‰) and sucrose (IAEA-CH_6_; δ^13^C = -10.4‰), were used for calibration of δ^13^C at a precision of 0.2‰. For nitrogen, international isotope secondary standards of known ^15^N/^14^N ratios in relation to air, namely: ammonium sulphate (IAEA N1; δ^15^N = +0.4‰ and IAEA N_2_; δ^15^N = +20.3‰) and potassium nitrate (IAEA NO_3_; δ^15^N = +4.7‰) were used for calibration of δ^15^N to a precision of 0.3‰. Atropine (70.56%C, 4.84%N) was used as a standard for elemental composition of C and N. The experimental precision based on the standard deviation of replicates of an atropine standard was 0.3% for both carbon and nitrogen. The reference materials used for the analysis are distributed by the International Atomic Energy Agency (IAEA). 

#### Oxygen

Dentine powder was pre-treated with 30% hydrogen peroxide for 24 hours to remove organics, then rinsed carefully with milli-Q water and treated with 1M calcium acetate /acetic acid buffer for another 24 hours to remove any diagenetic carbonate. Then it was rinsed again following the same procedure and dried for 24 hours. Stable isotopic determinations were carried out in 10 mg of dentine using a Finnigan-MAT 252 mass spectrometer, fitted with a Kiel Carbonate Device III (Thermo Electron - Dual Inlet) where samples were dissolved in 100% phosphoric acid at 70°C with concurrent cryogenic trapping of CO_2_ and H_2_O. The CO_2_ was then admitted to the mass spectrometer for analysis. NBS-19 international standard was used, with δ^18^O (in relation to V-PDB) = −2.20‰ values, certified by the IAEA. Analytical reproducibility (1σ parameter) obtained from replicate analyses of the powder fraction of reference material NBS-19 was better than ±0.08 for δ^18^O. Analyses were carried out in the laboratories of the University of Barcelona (Centres Científics i Teconlògics, CCiT-UB).

The natural abundances of ^13^C, ^15^N, and ^18^O are expressed as permil (‰) deviation from the standards as defined by the following equation [53]: 

$$\delta^{i}E=\left[R{\left({}^{i}E/{}^{j}E\right)}_{s}-R{\left({}^{i}E/{}^{j}E\right)}_{std}\right]/R{\left({}^{i}E/{}^{j}E\right)}_{std}$$

where R is the ratio of the heavy isotope (^i^E) to the light isotope (^j^E) of element E, in the sample (s) and in the standard (std). 

Because δ^18^O values in zoology are more commonly presented relative to standard mean ocean water (SMOW) and, to allow comparison with published data, δ^18^O values were converted from V-PDB to SMOW using the following equation [54]:

$$\delta^{18}O_{SMOW}=1.03086\times \delta^{18}O_{V-PDB}+30.86$$

### Statistical analysis

Data were tested for normality with a Kolmogorov-Smirnov test of goodness of fit and homogeneity of variances with the Levene’s test. Because all δ values from both the NW Spain and the Denmark groups of samples showed a normal distribution (Z<0.84 and p>0.05), subsequent analyses were conducted with parametric tests. T-student test was used to compare means of δ^13^C, δ^15^N and δ^18^O between regions in the 3rd year dentinal GLG. 

To investigate the influence of age and sex in the distinction between the two regions, and the interactions among these factors, an ANCOVA with age as covariate and region and sex as fixed factors was performed. Moreover, as the age span of individuals varied markedly and the individuals from NW Spain were noticeably younger than those from Denmark, the analysis was restricted to the age-segment corresponding to the first 20 years of life. 

Age was observed to have a significant influence on the stable isotope values, so the stable isotope age-related trends in each individual were investigated through the application of both a general linear model (GLM) and a non-linear general additive model (GAM) with varying number of knots depending on the number of observations. The fit of the two models for each individual was evaluated through the comparison of their relative AIC (Akaike's Information Criterion) values.

Statistical calculations were carried out using the statistical package SPSS15 (SPSS Inc., Chicago, IL, USA), and the mgcv package in the R-3.0.2 software

## Results

Table S1 details the stable isotope values and the biological information available from each individual analysed. The C/N ratio of the tooth dentine ranged 2.8-3.4 (mean: 3; sd:± 0.11), values which are well within the range of unaltered collagen, thus indicating absence of contamination by lipids or other materials [55].

### Nitrogen isotopes

**Figure 2:**
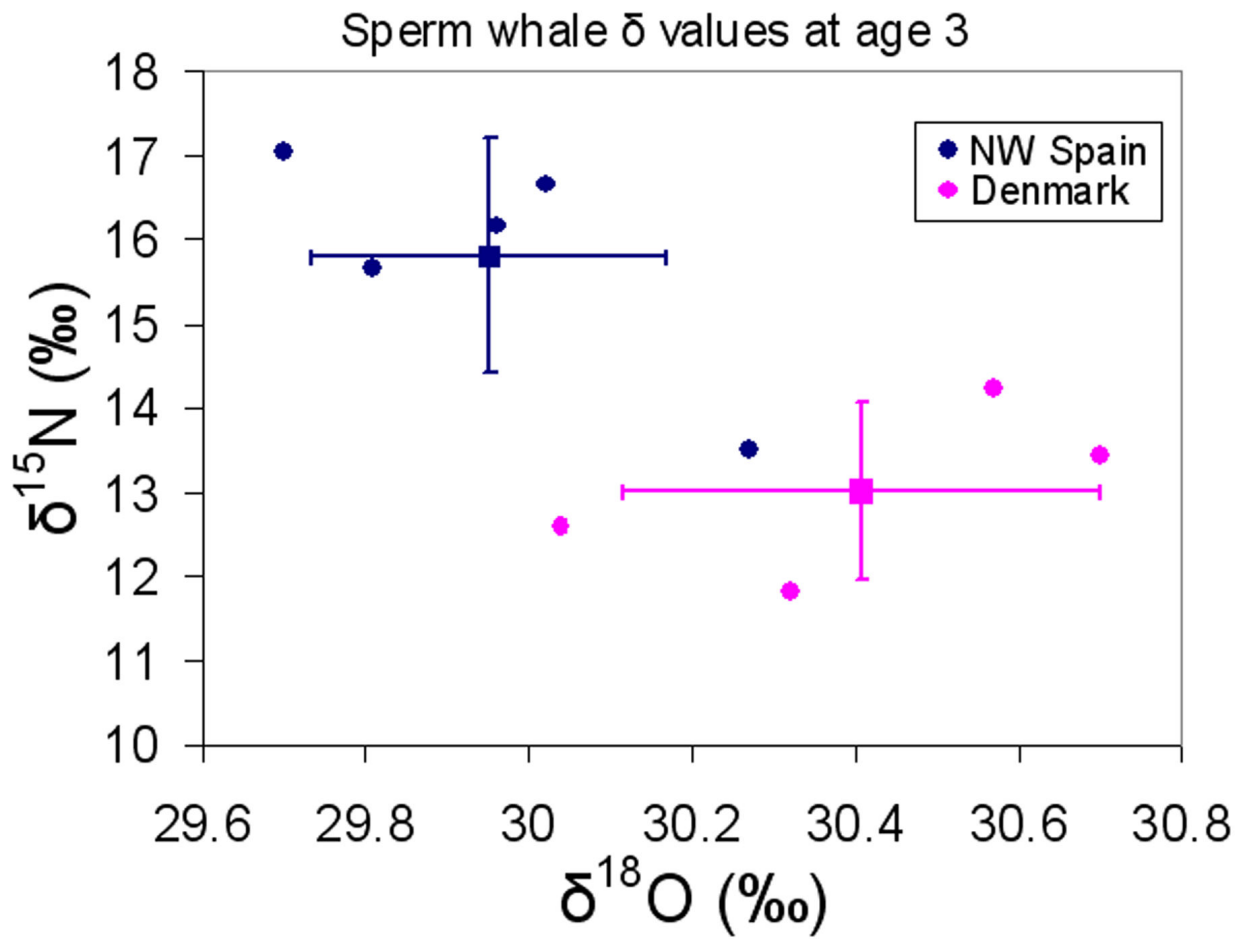
Comparison of δ^15^N and δ^18^O values (mean±sd) of the 3rd year dentinal growth layer group (GLG) in sperm whales sampled in Denmark (*n*=4) and NW Spain (*n*=5) (*p*<0.05 for δ^15^N; and *p*<0.01 for δ^18^O).

**Figure 3:**
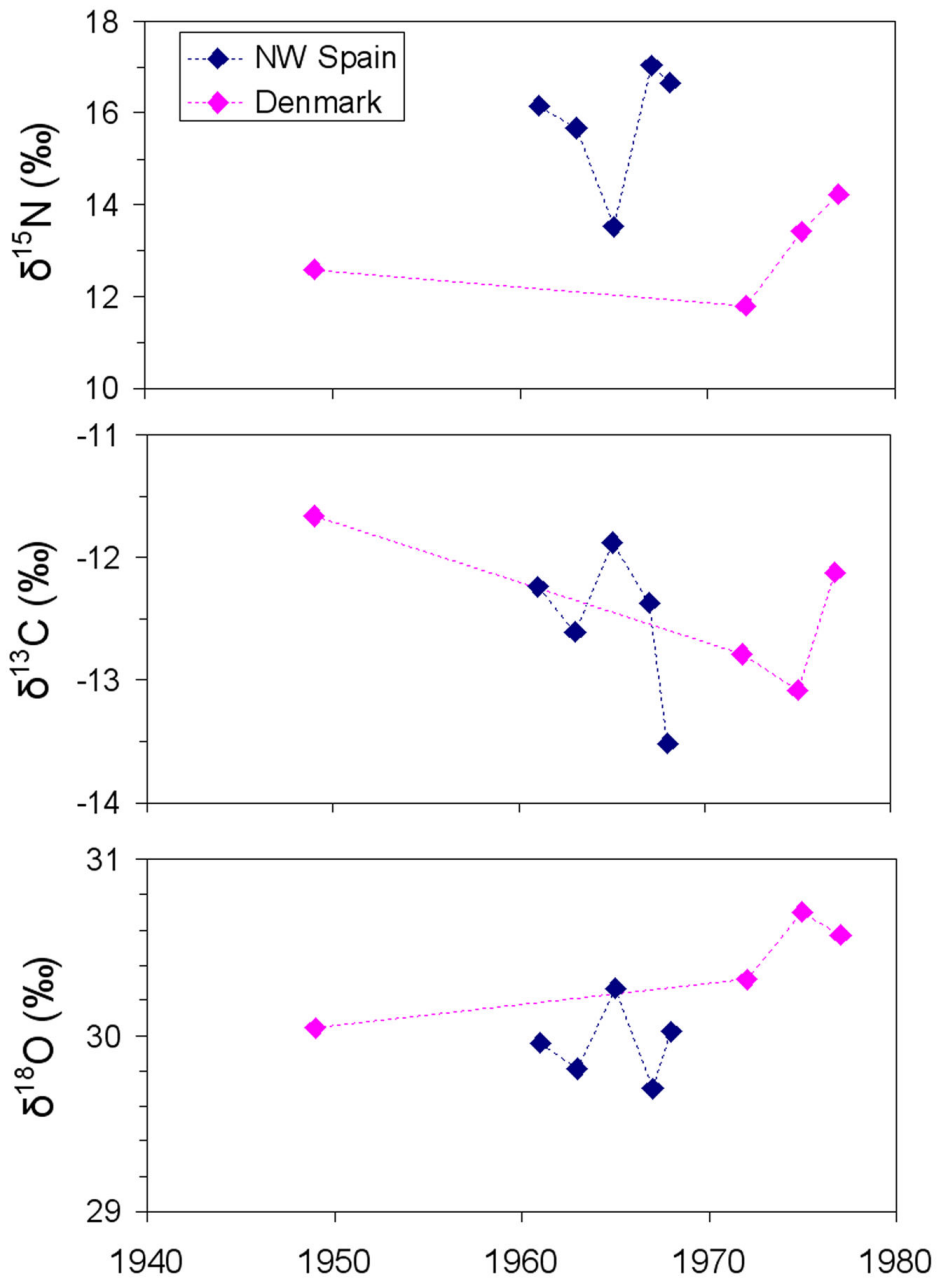
Relationship between δ^15^N, δ^13^C and δ^18^O values and the year of formation of the 3rd year dentinal growth layer group (GLG) in sperm whales from NW Spain and Denmark.

Specimens from NW Spain showed δ^15^N values ranging from 13.5‰ to 17.9‰ (mean:15.4‰; sd:±1.1) and those from Denmark ranging from 11.8‰ to 16.2‰ (mean:13.9‰; sd:±1.0). The δ^15^N in dentine deposited at 3 years of age was significantly lower in sperm whales from Denmark (mean:13.0‰; sd:±1.0) than in those from NW Spain (mean:15.8‰; sd:±1.4) (Table 2, Figure 2). To exclude the effect of potential variation in isotopic baselines with time, we examined the relationship between δ^15^N values and the year of formation of the 3rd year dentinal GLG in sperm whales from both regions and found absence of correlation (Figure 3a, Denmark: Pearson's r=-0.27, p=0.73; Spain: Pearson's r=-0.50, p=0.39).

**Table 2:** Statistical results of Independent samples test between stable isotope values of the 3rd year dentinal growth layer group (GLG) in sperm whales from NW Spain and Denmark.

	**Levene's test for equality of variances**			**t-test for equality of means**				
	F	Sig.		t	df	Sig. (2-tailed)	Mean difference	Std error difference
**δ^13^C**	0.17	0.69		0.26	7	0.80	0.11	0.42
**δ^15^N**	0.10	0.76		-3.33	7	0.01	-2.79	0.84
**δ^18^O**	0.67	0.44		2.68	7	0.03	0.46	0.17

Results from the ANCOVA indicated that region (F(1, 62)=18.63, p<0.001) and age (F(1, 62)=6.72, p<0.05) showed strong interaction with δ^15^N, while sex (F(1, 62)=3.80, p=0.056) was close to show interaction (Table S2). 

**Figure 4:**
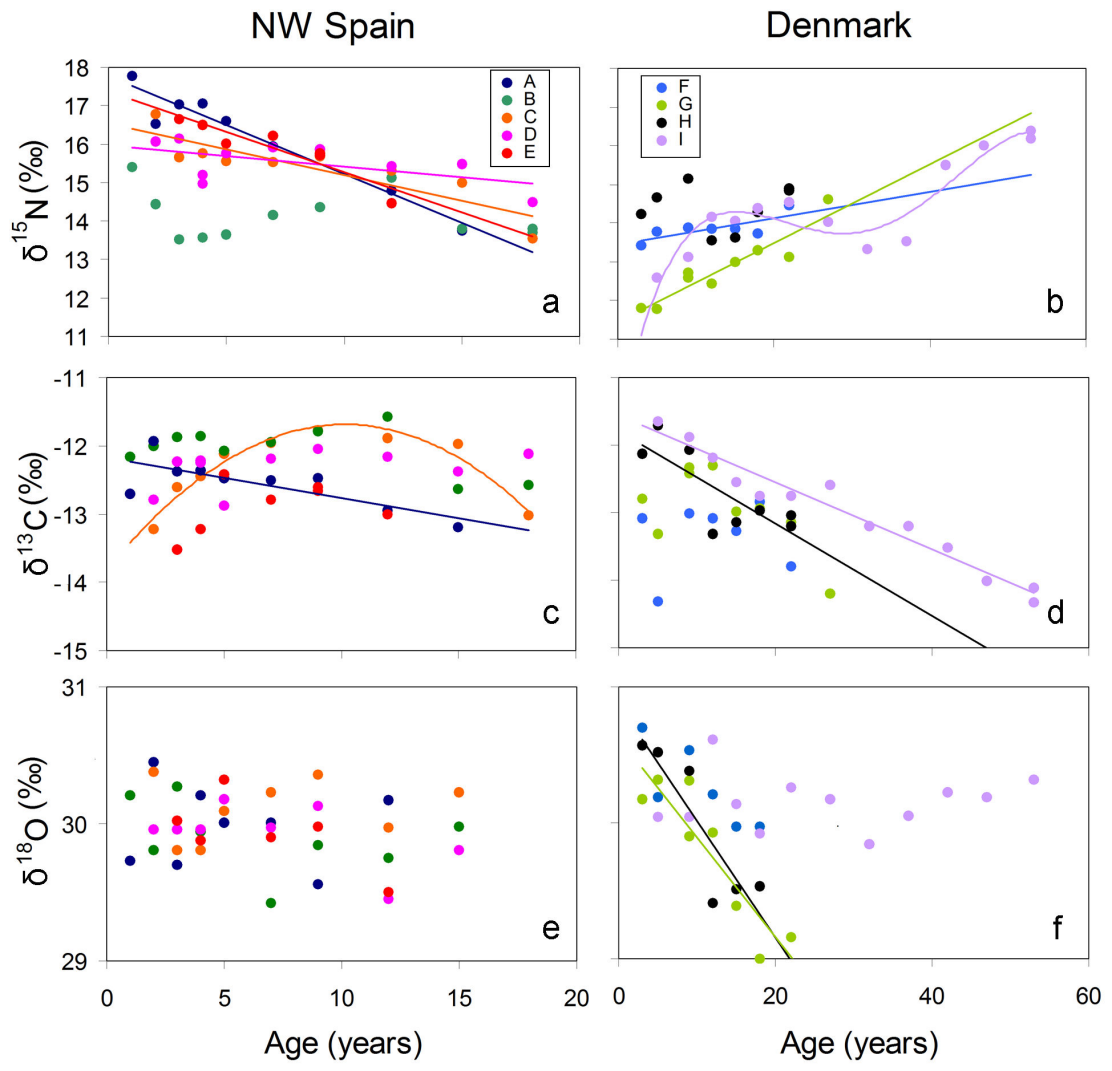
Relationship between stable isotope values (δ^15^N: a, b; δ^13^C: c, d and δ^18^O: e, f) and age in sperm whales sampled in Denmark and NW Spain. Each individual, identified as A-I according to details shown in Table 1, is represented by a different colour.

Trends of δ^15^N with age for each individual are depicted in Figures 4a and 4b, while AIC and p values resulting from the application of the GLM and GAM models to each individual set of data are shown in Table 3. Based on the results obtained from the application of the two models and the comparison of the relative AIC values, δ^15^N showed a significant positive linear correlation with age in two (F and G) out of the four males sampled in Denmark, while one of the other males from Denmark (I) showed a non-linear relationship and the other (H) showed a non-significant trend (Figure 4b). Regarding the five individuals from NW Spain, δ^15^N significantly decreased linearly with age in the three females (C, D and E) and in one male (A), while another male (B) showed a non-significant trend (Figure 4a). No substantial differences in the AIC values of the two models were found in any of the NW Spain individuals (Table 3).

**Table 3:** AIC and p values resulting from the application of the linear GLM and non-linear GAM models to each individual set of data to investigate the correlation between stable isotope values and age.

		**GLM**		**GAM**	
	**ID**	**AIC**	**p**	**AIC**	**p**
**δ^15^N**	**A**	11.47	<0.001	11.47	<0.001
	**B**	24.46	0.61	24.46	0.61
	**C**	15.28	<0.01	15.28	<0.01
	**D**	12.02	<0.05	12.02	<0.05
	**E**	7.01	<0.001	6.86●	<0.001
	**F**	4.39	<0.05	4.39	<0.05
	**G**	9.40	<0.001	9.40	<0.001
	**H**	17.19	0.90	17.19	0.90
	**I**	31.66	<0.001	18.11●	<0.001
**δ^13^C**	**A**	4.71	<0.05	4.71	<0.05
	**B**	8.26	0.17	3.47●	0.07
	**C**	17.88	0.64	-0.83●	<0.001
	**D**	6.97	0.24	6.97	0.24
	**E**	9.91	0.53	9.21●	0.69
	**F**	15.70	0.82	15.70	0.82
	**G**	15.90	0.13	10.83●	0.62
	**H**	10.87	<0.05	10.86●	<0.05
	**I**	-0.09	<0.001	-0.09	<0.001
**δ^18^O**	**A**	8.84	0.98	8.84	0.98
	**B**	5.92	0.42	5.49●	0.60
	**C**	4.19	0.64	4.19	0.64
	**D**	-0.92	0.10	-0.92	0.10
	**E**	2.09	0.14	2.09	0.14
	**F**	1.47	0.09	1.47	0.09
	**G**	5.78	<0.05	5.78	<0.05
	**H**	5.53	<0.05	5.53	<0.05
	**I**	1.45	0.69	1.45	0.69

● indicates a lower AIC value resulting from the application of the GAM model than that of GLM.

### Carbon isotopes

δ^13^C values in whales from NW Spain ranged from -13.6‰ to -11.6‰ (mean:-12.4‰; sd:±0.5), whereas in those from Denmark ranged from -14.3‰ to -11.7‰ (mean:-12.9‰; sd:± 0.7). No significant difference was found in dentine deposited at 3 years of age between sperm whales from Denmark (mean:-12.4‰; sd:±0.6) and NW Spain (mean:-12.6‰; sd:± 0.3) (Table 2). No correlation was found between δ^13^C values and the year of formation of the 3rd year dentinal GLG in the sperm whales from any of the regions (Figure 3b. Denmark: Pearson's r=0.78, p=0.22; Spain: Pearson's r=-0.14, p=0.82).

Results from the ANCOVA indicated that region (F(1, 62)=6.01, p<0.05) showed strong interaction with δ^13^C, while sex (F(1, 62)=1.40, p=0.241) and age (F(1, 62)=0.29, p=0.593) did not show interaction (Table S2). 

Trends of δ^13^C with age for each individual are depicted in Figures 4c and 4d, while AIC and p values resulting from the application of the GLM and GAM models to each individual set of data are shown in Table 3. δ^13^C showed a significant negative linear correlation with age in two (H and I) out of the four males from Denmark, while none of the other males from Denmark showed any significant trend (F and G) (Figure 4d). Regarding the five individuals from NW Spain, δ^13^C showed a significant negative linear correlation with age in one male (A) and a significant non-linear correlation in a female (C). In the other three individuals (B, D and E), no significant δ^13^C trends were detected (Figure 4c). In these three individuals, the AIC values resulting from the application of the GAM model were lower than those resulting from the GLM model (Table 3).

### Oxygen isotopes

δ^18^O values in whales from NW Spain ranged from 27.9‰ to 30.5‰ (mean:29.8‰; sd:±0.5), whereas in whales from Denmark ranged from 28.2‰ to 30.7‰ (mean:29.9‰; sd:±0.6). A significant difference was found in the dentine deposited at 3 years of age between sperm whales from Denmark (mean:30.4‰; sd:±0.3) and NW Spain (mean:30.0‰; sd:± 0.2) (Table 2, Figure 2). No correlation was found between δ^18^O values and the year of formation of the 3rd year dentinal GLG in the sperm whales from any of the regions (Figure 3c, Denmark: Pearson's r=-0.84, p=0.16; Spain: Pearson's r=0.65, p=0.23).

Results from the ANCOVA indicated that region (F(1, 58)=6.16, p<0.05) and age (F(1, 58)=16.23, p<0.001) showed strong interaction with δ^18^O, while sex (F(1, 58)=0.7, p=0.405) did not show interaction (Table S2). 

Trends of δ^18^O variations with age for each individual are depicted in Figures 4e and 4f, while AIC and p values resulting from the application of the GLM and GAM models to each individual set of data are showed in Table 3. δ^18^O significantly linearly decreased in two (G and H) out of the four males sampled in Denmark, while it did not show any trend in the remaining two (Figure 4f) individuals from Denmark, nor in the five individuals from NW Spain (Figure 4e). The AIC values calculated in the two models were nearly similar for all individuals (Table 3). 

## Discussion

The goal of the present study was to explore the utility of stable isotopes to investigate the occurrence of structure in the eastern North Atlantic population of sperm whales. More specifically, through the examination of individual age-related variation of stable isotope values in the GLGs in teeth we sought to determine potential segregation in breeding areas and/or dissimilarities in long-range movements throughout life-span. 

The departing hypothesis was that, if the studied individuals belonged to the same population, females and young individuals of both sexes would stay at the same breeding grounds, while adult males would migrate towards northern latitudes during at least part of their cycle. Because the isotopic signal of the environment is permanently recorded in the teeth dentine deposited each season [33-35], if the hypothesis were true we anticipated that the isotopic values of the GLGs corresponding to the first years of life would be similar in males and females, while those deposited at older ages would differ. We considered the GLG formed at age 3 as the best indication of the isotopic signal of the breeding grounds because at that age sperm whales have already been usually weaned but have not yet abandoned the breeding school [8,12,50]. The investigation had necessarily to focus on a limited number of individuals because in a long-lived species like the sperm whale the detailed study of age-related trends requires extensive sub-sampling of GLGs. Indeed, the sample size used here is well within the range of previous similar studies conducted on this and other comparable long-lived species [33,34,56]. 

Individuals from Denmark were all males, as expected given that only sperm whales of this gender occupy waters situated north of 40-45°N, while individuals from NW Spain belonged to both sexes (3 females and 2 males), again as expected because this region is within the latitudinal range of occurrence of the breeding schools [7,14]. Irrespectively of their gender, at age 3 individuals from Denmark showed lower δ^15^N, similar δ^13^C and higher δ^18^O than those from NW Spain (Table 2, Figure 2). The high variability among individuals from the same region (see Figure 2) could be due to shifts in maternal foraging areas by year or time period [22]. Because individuals had been sampled in different years and their total age was very variable, the year corresponding to age 3 differed greatly. To exclude the effect of potential variation in isotopic baselines with time, we examined the relationship between δ^15^N, δ^13^C and δ^18^O values and the year of formation of the 3rd year dentinal GLG in sperm whales from both regions and found absence of trend (Figure 3). Environmental factors such as temperature, light intensity and nutrient concentration are known to affect the δ^18^O, δ^13^C and δ^15^N values of primary producers [57] and therefore the dissimilarities in these profiles indicate that the geographical areas where the individuals had been born presented substantially different environmental characteristics and, very likely, were distant. 

However, the difference between sperm whales sampled in Denmark and in NW Spain was not apparently restricted to the early period of life. Thus, the ANCOVA test also demonstrated significant differences during the first 20 years, the segment of lifespan that was represented in the two regions. Most males from Denmark showed a significant increase, either linear or quadratic, in δ^15^N values with age (Figure 4b). Conversely, in NW Spain one male and all females showed a significant decreasing trend (Figure 4a). Previous studies [33,34] have shown considerable variation in both values and trends between individuals. However, δ^15^N in males usually tended to increase with age, as is the case in individuals from Iceland and Scotland [33,34]. This trend is attributed to the increasing strength and diving capacity that individuals acquire when growing old, characteristics that allow them to progressively feed on bigger prey, mostly giant squids, situated at higher trophic levels [8,58-60]. Why that trend was observed in most males from Denmark but the two males from NW Spain showed either no trend or a decreasing trend is unclear but again the difference is suggestive of dissimilarities between individuals from the two regions, either on their foraging behaviour or in their patterns of movements and, consequently, on the δ^15^N baselines of the waters they inhabit. Indeed, other males previously analysed from the Mediterranean and from India also showed age-related decreasing trends in their δ^15^N values [33]. On the other hand, the decreasing trend in δ^15^N values found in the females from NW Spain is probably explained by the fact that after the onset of reproductive activity they are likely to reduce the depth of their dives both during pregnancy and lactation and therefore to shift to shallower prey [9,10]. This is confirmed by previous analyses on another female from NW Spain which did not show any age-related δ^15^N trend and on two other females, one from the Azores and another from the Mediterranean Sea, that did show a decreasing trend [33]. 

δ^13^C values were also highly variable between individuals but again showed significant differences between regions (Figures 4c and 4d). In males, values decreased in about half of the sample both in Denmark and in NW Spain, as has been previously found to occur in individuals from Scotland [34], while the other half showed no definite trend as seen in whales from other regions [33]. In the females from NW Spain, values of one individual showed a non-linear correlation with age, while those of the other two individuals did not show any trend, again as previously observed elsewhere [33]. The high variability observed in all sample groups probably reflected periodic or seasonal changes in diet composition or in movements, like shifting from inshore to offshore waters. In particular, published carbon isoscapes suggest that the age-related decrease in δ^13^C values found in Denmark males (Figure 4d) could reflect a tendency to move to higher latitudes when they grow older [30,34,61]. 

As expected in a marine species [39], variability of δ^18^O values was small but still displayed substantial inter-annual fluctuation, the pattern of which differed between individuals. Two males from Denmark displayed a decreasing trend of δ^18^O with age (Figure 4f) but neither the other males from the same region nor any individual from NW Spain, irrespective of its gender, showed any trend (Figure 4e). Because δ^18^O tends to be depleted in higher latitudes and in colder water [41,62], the decrease found in the former group of males is likely to reflect migration to higher latitude or an increasing tendency to consume deeper, colder water prey as the individual grows old. 

Overall, the differences observed in C, N and O stable isotopes and trends point towards the existence of heterogeneities in the habitat used by sperm whales from Denmark and NW Spain, particularly in the location of their breeding grounds. This suggests the occurrence of structure in the eastern North Atlantic population of the species. 

Given that currently only one single management unit of the species is recognized for the whole North Atlantic [16,17], further research is needed to deepen into such potential structuring and, if occurring, to define the actual borders of any subpopulation.

## Supporting Information

Table S1 **Individual isotope values and biological information.**
(XLS)

Table S2 **Statistical results of One-way Analysis of Covariance (ANCOVA) with isotope values as dependent variable and age as covariate and region and sex as fixed factors.**
(DOC)
