# Supplementary material for: Stable Isotopes Provide Insight into Population Structure and Segregation in Eastern North Atlantic Sperm Whales
Source: PLoS One. 2013 Dec 6;8(12):e82398. doi: 10.1371/journal.pone.0082398 (PMC3855748; doi:10.1371/journal.pone.0082398)
Supplement: Table S2 — Statistical results of One-way Analysis of Covariance (ANCOVA) with isotope values as dependent variable and age as covariate and region and sex as fixed factors. (DOC) [file pone.0082398.s002.doc]

| **Tests of Between-Subjects Effects** | **Source** | **Typo III sum of squares** | **df** | **Mean square** | **F** | **Sig.** | **Partial Eta^2^** |
| --- | --- | --- | --- | --- | --- | --- | --- |
|  | Corrected model | 60.02 | 3 | 20.01 | 21.77 | 0.000 | 0.51 |
| **D.t Variable: δ^15^N** | Intercept | 3710.96 | 1 | 3710.96 | 4038.86 | 0.000 | 0.98 |
|  | Age | 6.18 | 1 | 6.18 | 6.72 | 0.012 | 0.10 |
| R^2^ = 0.513 | Region | 17.12 | 1 | 17.12 | 18.63 | 0.000 | 0.23 |
| (Adjusted R^2^ = 0.489) | Sex | 3.49 | 1 | 3.49 | 3.80 | 0.056 | 0.06 |
|  | Regin * sex | 0.00 | 0 |  |  |  | 0.00 |
|  | Error | 56.97 | 62 | 0.92 |  |  |  |
|  | Total | 14456.92 | 66 |  |  |  |  |
|  | Corrected Total | 116.98 | 65 |  |  |  |  |
|  | Corrected model | 2.11 | 3 | 0.70 | 2.56 | 0.063 | 0.11 |
| **D.t Variable: δ^13^C** | Intercept | 2497.52 | 1 | 2497.52 | 9112.66 | 0.000 | 0.99 |
|  | Age | 0.08 | 1 | 0.08 | 0.29 | 0.593 | 0.00 |
| R^2^ = 0.110 | Region | 1.65 | 1 | 1.65 | 6.01 | 0.017 | 0.09 |
| (Adjusted R^2^ = 0.067) | Sex | 0.38 | 1 | 0.38 | 1.40 | 0.241 | 0.02 |
|  | Regin * sex | 0.00 | 0 |  |  |  | 0.00 |
|  | Error | 16.99 | 62 | 0.27 |  |  |  |
|  | Total | 10354.34 | 66 |  |  |  |  |
|  | Corrected Total | 19.10 | 65 |  |  |  |  |
|  | Corrected model | 1.57 | 3 | 0.52 | 5.76 | 0.002 | 0.23 |
| **D.t Variable: δ^18^O** | Intercept | 12904.88 | 1 | 12904.88 | 141918.78 | 0.000 | 1.00 |
|  | Age | 1.48 | 1 | 1.48 | 16.23 | 0.000 | 0.22 |
| R^2^ = 0.229 | Region | 0.56 | 1 | 0.56 | 6.16 | 0.016 | 0.10 |
| (Adjusted R^2^ = 0.19) | Sex | 0.06 | 1 | 0.06 | 0.70 | 0.405 | 0.01 |
|  | Regin * sex | 0.00 | 0 |  |  |  | 0.00 |
|  | Error | 5.27 | 58 | 0.09 |  |  |  |
|  | Total | 55815.94 | 62 |  |  |  |  |
|  | Corrected Total | 6.84 | 61 |  |  |  |  |

Table S2: Statistical results of One-way Analysis of Covariance (ANCOVA) with isotope values as dependent variable and age as covariate and region and sex as fixed factors.
